# Maximum strength development in martial arts and perception and awareness: new approach for training methods

**DOI:** 10.3389/fspor.2025.1676250

**Published:** 2025-11-24

**Authors:** Giovanni Esposito, Sara Aliberti, Rosario Ceruso, Antonio Tessitore, Gaetano Raiola

**Affiliations:** 1 Research Center of Physical Education and Exercise, University of Pegaso, Naples, Italy; 2 Department of Neuroscience, Biomedicine and Movement Sciences, University of Verona, Verona, Italy; 3 Department of Movement, Human and Health Sciences, University of Rome “Foro Italico”, Rome, Italy

**Keywords:** combat sports, qualitative-quantitative connections, psychological aspects, performance aspects, strength analysis

## Abstract

The development of strength in combat sports is often studied independently of athletes' perceptions, despite its application in variable “open skills” contexts. While the literature highlights the impact of strength training on performance, limited research examines the link between strength improvement and athletes' awareness of associated psychophysical benefits. This study aimed to provide preliminary evidence exploring the relationship between the physical effects of a strength training protocol and athletes' perceptions and awareness. Twelve male Wushu Sanda athletes (16–27 years) with similar athletic characteristics participated. Pre-, mid-training, and post-testing measured one-repetition maximum (1RM) for Bench Press, Back Squat, and Deadlift, while questionnaires assessed perception and awareness. The training protocol consisted of 3 weekly sessions over 8 weeks. Statistical analysis (repeated-measures ANOVA/Friedman and Bonferroni/Wilcoxon *post hoc* tests) revealed significant strength gains and progressive improvements in confidence and awareness of training benefits. Quantitatively, participants showed increases of 17.3% in Bench Press, 14.7% in Back Squat, and 15.7% in Deadlift (*p* < 0.05). Differences were most pronounced between pre- and post-tests, underscoring the value of extended training. The findings suggest the effectiveness of the protocol in developing maximal strength and demonstrate a clear connection between physical improvements and enhanced athlete perception and awareness. Given the small sample size and lack of a control group, these findings should be interpreted with caution. These results highlight the importance of integrating psychological and motivational factors into physically demanding training programs to maximize outcomes.

## 1 Introduction

The development of strength is often analyzed in isolation, without adequately considering its relationship with psychological and perceptual factors associated with physical improvement. This limited approach may overlook how physical, cognitive, and emotional components interact to determine overall performance outcomes. In combat sports such as Wushu Sanda, strength is expressed through “open skills” that require athletes to adapt constantly to dynamic and unpredictable environments (1). Unlike “closed skills,” which occur in controlled and predictable settings, open skills demand continuous decision-making, adaptability, and situational awareness. These cognitive and perceptual abilities interact closely with the physical component, shaping athletes' capacity to execute technical actions effectively under pressure. Combat sports are therefore classified as situational disciplines, in which both quantitative aspects of physical power and qualitative dimensions of motor control are essential for success. Strength and power provide the foundation for performing techniques with precision and speed, but their effective application relies on neuromuscular coordination, anticipatory abilities, and cognitive processing (2, 3). Research has emphasized that strength training combined with situational drills enhances athletes' ability to translate raw power into functional, competition-specific movements (4). Furthermore, improving neuromuscular efficiency contributes not only to better technical execution but also to injury prevention through greater joint stability and muscle coordination (5, 6).

Recent approaches to physical preparation have increasingly adopted individualized and context-specific methodologies, encouraging athletes to play an active role in their psychophysical development. Within this perspective, the Ecological-Dynamics framework views training as an interactive process between the athlete and the environment, fostering perceptual–motor coupling and facilitating skill transfer to competition (7, 8). Similarly, integrating cognitive and psychological components, such as mindfulness or reaction-based drills, has been shown to enhance attention, focus, and reaction time (9–11). Although numerous studies have explored the physical effects of strength training in combat sports (12–15), there is limited evidence on how improvements in strength influence athletes' self-perception and awareness of psychophysical benefits. This research gap is particularly relevant in *Wushu Sanda*, where success depends on the dynamic integration of physical power, motor control, and psychological readiness.

The present study aimed to investigate the relationship between the physical effects of a structured strength training program and athletes' self-perception and awareness in Wushu Sanda. Specifically, it examined how increased strength levels affected athletes' perceptions of body awareness and confidence in the effectiveness of their training. It was hypothesized that the proposed strength training protocol would lead to improvements in maximal strength performance and to enhanced self-perceived psychophysical awareness among the athletes. By addressing the interaction between physical and psychological domains, this study seeks to contribute to a more holistic understanding of performance development in combat sports, offering practical insights for coaches and practitioners involved in high-level athlete preparation.

## 2 Methods

### 2.1 Design and participants

This study employed a pre-post research design without a control group, chosen to explore the feasibility and impact of a strength training protocol within a realistic sports practice context. While this design allowed for the assessment of changes observed during the intervention period, it does not completely exclude potential con-founding factors, such as natural strength development, external physical activities, or placebo effects. The absence of a control group was primarily due to logistical constraints related to the limited number of available competitive athletes within the same training center. In addition, the aim of this preliminary study was to explore the feasibility and short-term impact of the proposed protocol rather than to establish causality. Future studies incorporating randomized control groups will be necessary to strengthen internal validity and confirm these findings. The convenience sample consisted of 12 male Wushu Sanda athletes aged 16–27 years, selected for their similar physical and athletic characteristics, as well as their optimal psychophysical condition. Anthropometric characteristics were recorded at baseline. Participants had a mean body weight of 70.8 ± 6.3 kg, height of 174.5 ± 5.2 cm, and body mass index (BMI) of 23.2 ± 1.8 kg/m^2^. These measures were taken using a calibrated digital scale and stadiometer under standardized conditions. Inclusion criteria included: (1) male athletes aged between 16 and 27 years; (2) a minimum of 5 years of continuous Wushu Sanda training experience; (3) regular participation in regional or national-level competitions; (4) medical clearance for high-intensity strength training. Exclusion criteria were: (1) recent musculoskeletal injury (within 6 months); (2) cardiovascular or metabolic disorders; (3) use of performance-enhancing drugs or supplements during the intervention. The study was conducted between February and April 2024 in a combat sports training facility located in Salerno, Italy, equipped with Olympic-standard weightlifting stations and monitoring tools for data collection. This age range was chosen to encompass individuals in an active phase of athletic and psychological development, which is critical for improving strength and body awareness. Furthermore, all participants had a minimum of 5 years of experience in Wushu Sanda, a factor that mitigates the potential influence of age-related differences on the results. Importantly, the chosen age range reflects the demographic composition of athletes commonly engaged in competitive contexts for this discipline. The sample size (*n* = 12) was determined based on *a priori* power analysis (G*Power 3.1), assuming a medium effect size (*f* = 0.35), *α* = 0.05, and power (1−*β*) = 0.80 for repeated-measures ANOVA across three time points, which indicated a minimum sample of 10 participants. Participant adherence to the 8-week training protocol was closely monitored, and all 12 participants successfully completed the program without any dropouts. Compliance was high, with no major deviations from the prescribed protocol. Minor challenges, such as scheduling conflicts and initial adjustments to the intensification phase, were reported but did not significantly impact the overall outcomes. These observations highlight the im-portance of designing protocols that accommodate individual needs and contexts. All training sessions were supervised by a certified Wushu Sanda coach, with oversight by a researcher to ensure adherence to the intervention protocol. Each training session included a 15-minute standardized warm-up consisting of dynamic stretching and joint mobility exercises, followed by a 10-minute cool-down and recovery period involving light aerobic activity and static stretching, ensuring consistent preparation and recovery across participants.

### 2.2 Data collection

At the beginning of the study, the one-repetition maximum (1RM) for the Back Squat, Bench Press, and Deadlift was assessed for each athlete to establish baseline strength levels. The assessments were performed over two non-consecutive testing sessions to minimize fatigue effects. Prior to testing, participants completed a standardized warm-up consisting of 5 min of light aerobic activity followed by dynamic stretching and progressive sets of each exercise with submaximal loads (approximately 50%, 70%, and 85% of the estimated 1RM). For each lift, the load was progressively increased until the participant was unable to complete a full repetition with correct technique. The heaviest successfully lifted load was recorded as the 1RM value, expressed in kilograms (kg). Adequate rest intervals of 3–5 min were provided between maximal attempts to ensure full recovery. All tests were conducted on-site at the training facility under the supervision of the research team, following standardized testing protocols and using calibrated Olympic barbells and weight plates to ensure measurement reliability and validity (16).

Finally, participants administered questionnaires specifically designed to assess their perception and awareness at pre-, mid-training, and post-training stages. The questionnaire consisted of 9 questions, with responses rated on a 5-point Likert scale, ranging from 1 (strongly disagree) to 5 (strongly agree). Content validity was established through expert review: three sport science specialists and two experienced Wushu coaches independently evaluated each item for clarity, relevance, and representativeness of the intended constructs (body control, strength perception, and awareness of strength importance). Minor adjustments in wording were made based on their feedback to ensure conceptual accuracy and comprehensibility for the athlete population. A detailed description of the questionnaire is presented in Table 1.

**Table 1:** Perception-awareness questionnaire.

Time	Questions
Pre	(Q1) Do you feel confident in your ability to manage your body effectively?
	(Q2) Do you think the program can improve your strength levels?
	(Q3) How aware are you of the importance of strength in combat?
Mid-training	(Q1a) How much have you improved in managing and controlling your body?
	(Q2a) Does the program meet your expectations in terms of strength improvement?
	(Q3a) How aware are you of the importance of strength in combat?
Post	(Q1b) How much do you think you’ve improved in managing your body?
	(Q2b) Do you think the protocol has improved your ability to express strength?
	(Q3b) How aware are you of the importance of strength in combat?

Before using the questionnaire in the main study, a pilot test was conducted to assess its internal consistency. The pilot group included a smaller sample of athletes who underwent similar training and testing procedures. Cronbach's alpha, a widely accepted measure of internal consistency, was calculated for each dimension of the questionnaire. Cronbach's alpha values for the three dimensions were body control (*a* = 0.85), strength improvement (*a* = 0.88) and awareness of strength training's im-portance (*a* = 0.83). These values indicate high internal reliability for the questionnaire, suggesting that the items within each dimension are consistently measuring the in-tended construction.

### 2.3 Training protocol

Following the initial testing, athletes were assigned a training protocol (Table 2) to be performed 3 times a week for 8 weeks. The chosen protocol was the Russian strength training protocol, which consists of two main phases: the accumulation phase and the intensification phase. In each week, intensity increased by 5%, while volume decreased. This training approach was selected based on its proven efficacy in eliciting maximal strength adaptations through progressive overload and systematic load variation, as demonstrated in previous literature on strength periodization (16). The Russian model has been widely applied in combat and strength sports due to its structured balance between volume and intensity, promoting neural and muscular adaptations relevant to performance in Wushu Sanda.

**Table 2:** Training protocol.

Week	Monday	Wednesday	Friday
1	Squat 6 × 3 (80% 1RM)	Deadlift 6 × 3 (80%1RM)	Bench press 6 × 3 (80%1RM)
	Bench press 6 × 2 (80% 1RM)		Squat 6 × 2 (80% 1RM)
2	Squat 6 × 4 (80%1RM)	Deadlift 6 × 4 (80%1RM)	Bench press 6 × 4 (80%1RM)
	Bench press 6 × 2 (80%1RM)		Squat 6 × 2 (80% 1RM)
3	Squat 6 × 5 (80%1RM)	Deadlift 6 × 5 (80%1RM)	Bench press 6 × 5 (80%1RM)
	Bench press 6 × 2 (80%1RM)		Squat 6 × 2 (80% 1RM)
4	Squat 6 × 6 (80%1RM)	Deadlift 6 × 6 (80%1RM)	Bench press 6 × 6 (80%1RM)
	Bench press 6 × 2 (80%1RM)		Squat 6 × 2 (80% 1RM)
5	Squat 5 × 5 (85%1RM)	Deadlift 5 × 5 (85%1RM)	Bench press 5 × 5 (85%1RM)
	Bench press 6 × 2 (80%1RM)		Squat 6 × 2 (80% 1RM)
6	Squat 4 × 4 (90%1RM)	Deadlift 4 × 4 (90%1RM)	Bench press 4 × 4 (90%1RM)
	Bench press 6 × 2 (80%1RM)		Squat 6 × 2 (80% 1RM)
7	Squat 3 × 3 (95%1RM)	Deadlift 3 × 3 (95%1RM)	Bench press 3 × 3 (95%1RM)
	Bench press 6 × 2 (80%1RM)		Squat 6 × 2 (80% 1RM)
8	Squat 2 × 2 (100%1RM)	Deadlift 2 × 2 (100%1RM)	Bench press 2 × 2 (100%1RM)
	Bench press 6 × 2 (80%1RM)		Squat 6 × 2 (80% 1RM)
9	Squat 1 × 105%1RM	Deadlift 1 × 105%1RM	Bench press 1 × 105%1RM

The accumulation phase, consisting of the first 4 weeks, alternated between heavy and light training sessions for the different exercises. When the squat session was heavy, the bench press would be light (6 × 2 at 80% 1RM), and vice versa. The deadlift was always trained on its own with volume increases. The first day of Week 1 included heavy squats at 80% 1RM with a 6 × 3 scheme, with 2 min of rest between sets. This was followed by light bench press (6 × 2 at 80% 1RM). Day 2 focused on deadlifts with a 6 × 3 scheme at 80% 1RM, and on Day 3, heavy bench press (6 × 3 at 80% 1RM) was paired with light squats (6 × 2 at 80% 1RM). Over the first 4 weeks, an additional set was added to each heavy lift every week, eventually reaching a 6 × 6 scheme. The final 4 weeks (intensification phase) saw a 5% increase in intensity and a reduction in volume. On Week 5, Day 1 involved heavy squats (5 × 5 at 85% 1RM) with light bench press (6 × 2 at 80% 1RM), while Day 2 focused on heavy deadlifts (5 × 5 at 80% 1RM). Day 3 included heavy bench press (5 × 5 at 85% 1RM) and light squats (6 × 2 at 80% 1RM). For the heavy lifts, the progression was as follows: Week 5 used 5 × 5, Week 6 used 4 × 4, Week 7 used 3 × 3, and Week 8 used 2 × 2. The 1RM was retested during Week 9. The entire training program was carried out in a facility specializing in combat sports.

To minimize confounding factors, athletes were instructed to maintain their usual daily routines, avoid additional resistance or endurance training outside the protocol, and report any unplanned physical activities. Compliance with these instructions was monitored weekly through self-reported training logs. Nutrition was not manipulated experimentally; however, participants were required to maintain their habitual diet throughout the 8-week period. Nutritional adherence was informally monitored through weekly check-ins, during which athletes confirmed no significant dietary changes or use of ergogenic supplements.

Warm-up and recovery procedures were standardized across sessions: before each workout, athletes performed 10 min of general warm-up (light jogging and joint mobility drills) and specific warm-up sets for each exercise (two sets at 50% and 70% 1RM). Post-session recovery consisted of 5 min of low-intensity aerobic activity and static stretching of major muscle groups.

### 2.4 Statistical analysis

Data normality was verified using Shapiro Wilk test. The data analysis was descriptive and was subsequently supported by repeated measures ANOVA to investigate differences in each athlete at three different time points (pre, mid-training, post), followed by *post hoc* Bonferroni for multiple comparisons, for normally distributed quantitative data. The Friedman test, followed by the signed-rank test, was used for multiple comparisons of ordinal/qualitative data that did not meet the assumptions for parametric tests. Partial square Eta was calculated to determine the effect size for normal data and Kendall's *W* for non-normal data. Significance was set at *p* < 0.05. Data analyses were performed using the Statistical Package for Social Science software (IBM SPSS Statistics for Windows, version 25.0, IBM, SPSS Inc., Armonk, NY, USA).

## 3 Results

Table 3 presents the descriptive statistics for the one-repetition maximum (1RM) tests (squat, bench press, and deadlift) at three different time points: pre-test, mid-training, and post-test. The average of 1RM squat starts from 76.40, after 1 month to 79.17 until 87.60 kg. The average 1RM bench press test begins at 58.30 kg, increases to 61.46 kg after 1 month, and reaches 68.40 kg by the final assessment. Similarly, the average 1RM deadlift starts at 105.40 kg, rises to 110.33 kg after 1 month, and reaches 122.00 kg at the final evaluation. This table provides a clear over-view of the progression in participants' performance across the training period.

**Table 3:** Descriptive statistics.

Athlete	1RM squat (kg)			1RM bench press (kg)			1 RM deadlift (kg)		
	Pre	Mid-training	Post	Pre	Mid-training	Post	Pre	Mid-training	Post
1	70	72	82	52.5	55.5	65	95	98	110
2	67.5	69	78.5	50	56	62.5	90	95	107.5
3	75	79	86	55	57	67.5	107.5	110.5	125
4	82.5	85	90	62.5	64	71	115	120	132
5	77	80	85	57.5	59	62.5	110	117.5	127.5
6	65	67	73	50	54	62.5	87.5	89.5	100
7	90	93	98.5	72	75.5	80	127.5	130	140
8	80	82	90	62.5	64.5	71.2	112.5	119	127.5
9	75	79	85.5	57.5	59	65.7	105	114	122.5
10	62.5	63	75	50	55	61.2	85	92	102
11	105	110	130	78	81	90	137.5	140	155
12	67.5	71	77.2	52.5	57	61.5	92.5	98.5	115
Mean	76.40	79.17	87.60	58.30	61.46	68.40	105.40	110.33	122.00
Std. dev	11.98	12.85	15.18	9.03	8.58	8.73	16.34	15.95	16.15

**Figure 1:**
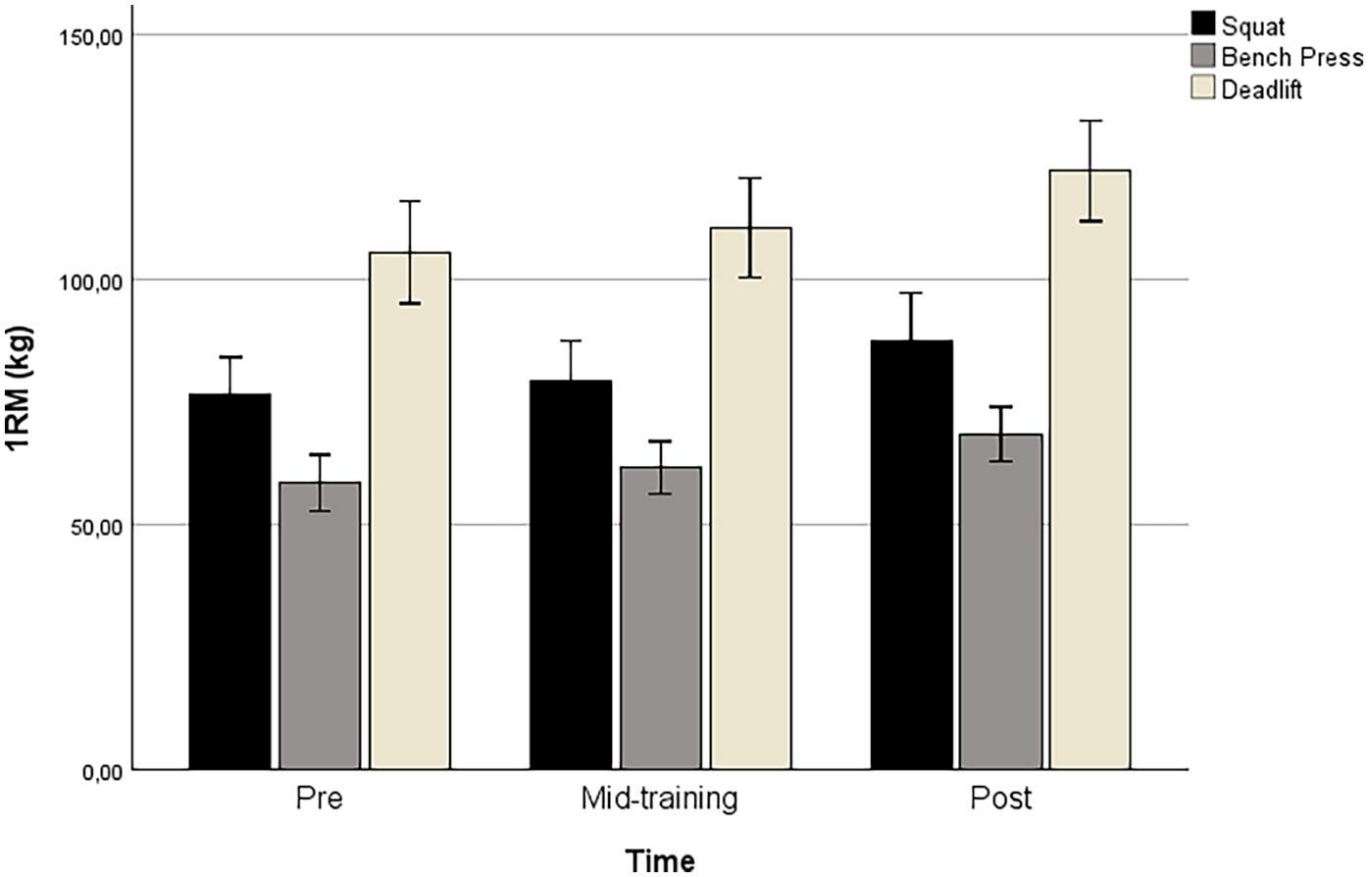
One-repetition maximum (1RM) strength changes in squat, bench press, and deadlift at pre, mid-training, and post assessments.

Table 4 presents the percentage changes in the mean performance values for the 1RM tests (squat, bench press, and deadlift) calculated across the three evaluation time points: pre-test, mid-training, and post-test. Specifically, it highlights the percentage variations between pre-test and mid-training, mid-training and post-test, and pre-test and post-test. These data provide insights into the relative improvement in performance for each exercise over the course of the training program. Figure 1 illustrates the changes in 1RM values across different time points for Squat, Bench Press, and Deadlift.

**Table 4:** Percentage changes in the mean values of 1RM tests.

Exercise	Pre-test mean	Mid-training mean	Post-test mean	% Change pre to mid	% Change mid to post	% Change pre to post
1RM squat	76.40	79.17	87.60	3.63%	10.65%	14.66%
1RM bench press	58.30	61.46	68.40	5.42%	11.29%	17.32%
1RM deadlift	105.40	110.33	122.00	4.68%	10.58%	15.75%

Table 5 provides the results of the repeated measures ANOVA, revealing significant changes in performance across the three time points for each test (squat, bench press, and deadlift), with *F*-values indicating strong effects [Squat: *F*_(2, 22)_ = 58.66, *p* < 0.001, *η*^2^*ₚ* = 0.91; Bench press: *F*_(2, 22)_ = 146.36, *p* < 0.001, *η*^2^*ₚ* = 0.85; Deadlift: *F*_(2, 22)_ = 273.61, *p* < 0.001, *η*^2^*ₚ* = 1.02]. The effect sizes indicate large practical significance across all exercises. *post-hoc* analyses, using pairwise comparisons, showed statistically significant improvements only between the pre-test and post-test values (*p* < 0.05).

**Table 5:** Repeated measures ANOVA and *post-hoc*.

Exercise	Comparison	Mean diff	*p*-value	Lower	Upper	Effect size (partial eta squared)
Squat	Mid-training vs. post	8.39	0.2888	−5.04	21.82	0.52
	Mid-training vs. pre	−2.75	0.8706	−16.18	10.68	0.17
	Post vs. pre	−11.14	0.0395	−24.57	−2.29	0.91
Bench press	Mid-training vs. post	6.93	0.1459	−1.87	15.72	0.60
	Mid-training vs. pre	−3.13	0.6617	−11.92	5.67	0.22
	Post vs. pre	−10.05	0.0223	−18.85	−1.25	0.85
Deadlift	Mid-training vs. post	11.67	0.1953	−4.51	27.84	0.71
	Mid-training vs. pre	−4.92	0.7382	−21.09	11.26	0.30
	Post vs. pre	−16.58	0.0435	−32.76	−0.41	1.02

Mean diff: mean difference between groups; *p*-value: *p*-value corrected for multiple comparisons; lower/upper: 95% confidence interval limits; effect size: partial eta squared.

Table 6 summarizes the descriptive statistics for participants' responses to the questionnaire, assessing their perceptions on body control, strength improvement, and awareness of the importance of strength training at the three points (pre, mid-training, and post). Most participants initially display low levels of perception and awareness (e.g., Q1 = 2.58; Q2 = 3.33; Q3 = 3.25), which tend to increase over time (Q1b = 3.75; Q2b = 3.91; Q3b = 3.91).

**Table 6:** Questionnaire answers and descriptive statistics.

Athlete	Pre—Q1	Mid-training- Q1a	Post—Q1b	Pre—Q2	Mid-training- Q2a	Post—Q2b	Pre—Q3	Mid-training- Q3a	Post—Q3b
1	2	3	4	3	3	4	3	4	4
2	3	3	4	4	4	4	3	3	3
3	2	3	3	2	3	3	3	3	4
4	3	3	4	3	4	4	4	4	4
5	3	4	4	4	4	4	3	4	4
6	3	4	4	4	4	4	3	3	4
7	2	3	3	3	3	4	3	4	4
8	2	3	3	3	4	4	3	4	4
9	3	3	4	4	4	4	4	4	4
10	3	4	4	4	4	4	4	4	4
11	3	3	4	3	4	4	3	4	4
12	2	3	4	3	4	4	3	4	4
Mean	2.58	3.25	3.75	3.33	3.75	3.91	3.25	3.75	3.91
Std. dev.	0.51	0.45	0.45	0.65	0.45	0.28	0.45	0.45	0.28

Table 7 displays the results of the Friedman test, which examined changes in questionnaire responses over time. Significant differences (*p* < 0.05) were found across the time points for body control, strength improvement and awareness on the importance of strength training (*p* < 0.05). Body control improves significantly across all stages, while perceptions of strength improvement and awareness show notable increases primarily between the pre-training and mid-training phases. Between the mid-training and post-training phases, the values remain relatively stable. Effect sizes (Kendall's *W*) indicate moderate to large effects for these measures (ranging from 0.58 to 0.88).

**Table 7:** Friedman test and Wilcoxon *post-hoc*.

Measure	Group 1	Group 2	Mean diff	*p*	Lower C.I.	Upper C.I.	Effect size (Kendall's *W*)
Body control: *F* = 19.16, *p* < 0.000	Mid-training	Post	0.50	0.0143	0.205	0.795	0.64
	Mid-training	Pre	−0.67	0.0047	−0.945	−0.388	0.72
	Post	Pre	−1.17	0.0005	−1.387	−0.946	0.88
Strength improvement: *F* = 10.33, *p* = 0.0057	Mid-training	Post	0.08	0.3173	−0.080	0.247	0.32
	Mid-training	Pre	−0.42	0.0253	−0.708	−0.125	0.58
	Post	Pre	−0.50	0.0143	−0.795	−0.205	0.65
Awareness on the importance of strength training: *F* = 13.00, *p* = 0.0015	Mid-training	Post	0.17	0.1573	−0.054	0.387	0.40
	Mid-training	Pre	−0.50	0.0143	−0.795	−0.205	0.65
	Post	Pre	−0.67	0.0047	−0.945	−0.388	0.72

## 4 Discussion

The results of the present study suggest a trend of improvement in the strength levels of the 12 athletes following the implemented 8-week training proto-cycle. Specifically, a significant tendency was observed between the pre-training and post-training phases, but the lack of a control group limits the possibility of attributing these changes solely to the training intervention. The descriptive analyses indicate a tendential increase in strength levels across the three key moments of the study. How-ever, the absence of statistically significant differences between the pre- and mid-training phases, as well as between the mid-training and post-training phases, highlights the need for cautious interpretation of the findings. These trends underscore the importance of prolonged and structured training in developing strength, particularly in combat sports, where such attributes are crucial for performance.

Percentage changes were calculated for each exercise (squat, bench press, and deadlift) to provide a clearer sense of the magnitude of the observed gains, alongside statistical significance values. On average, participants showed an average increase of 14.7% (from a mean of 76.4 to 87.6 kg, *p* < 0.05) regarding squat test, an average in-crease of 17.4% (from a mean of 58.3 to 68.4 kg, *p* < 0.05) for bench press, and an average increase of 15.7% (from a mean of 105.4 to 122.0 kg, *p* < 0.05) for deadlift. The data also revealed individual variations in strength gains, with percentage improvements ranging from 10% to 24% among the participants. For example, Participant 11 exhibited the highest gains in the squat (23.8%) and bench press (15.4%), reflecting consistent adherence and strong baseline proficiency. Conversely, Participant 10 demonstrated relatively modest gains in the deadlift (10%), possibly due to initial technical challenges during the intensification phase. The analysis of these variations suggests that while the training protocol was effective across the cohort, individual factors such as baseline strength, technical execution, and adaptability to the protocol may influence the magnitude of strength improvements. However, without a control group, these variations cannot be fully contextualized against external influences or natural progression.

Combat sports place a significant emphasis on the strength levels of athletes, a factor that greatly influences athletic performance. In this context, the implementation of the Russian cycle appears to be an effective method for developing maximum strength (17). This preliminary study suggested that the Russian method can generate significant improvements in the main weightlifting movements, results that are essential for enhancing athletes' ability to express strength efficiently and in a controlled manner. Moreover, increases in maximal strength have been associated with improvements in in-match performance, technical execution, and reduced risk of injury in combat athletes (6, 18). However, these results should be interpreted as preliminary evidence rather than confirmation of efficacy. Previous studies support these findings, highlighting the role of structured strength programs in improving both physical and motor skills in combat athletes (19, 20). In addition to improvements in strength levels, a significant increase was observed in the athletes' perception and awareness at the three key points of the study. It emerged that most athletes progressively improved their sense of security in controlling and effectively managing their bodies. This improvement was evident even in the early stages of the protocol, with a further significant increase between the initial and final phases. Regarding the perception of the program's usefulness in improving strength levels, the results show a significant increase between the pre and post phases. However, between the mid-training and post phases, the levels of perception remained relatively stable, suggesting that most athletes had already gained a clear awareness of the benefits of the protocol in the earlier stages of the study. Similarly, the awareness of the importance of strength in combat followed the same trend, with significantly higher values in the final phase compared to the initial phase. These findings align with established frameworks in sport psychology, such as Bandura's self-efficacy theory, which suggests that perceived competence and confidence in one's abilities are crucial for sustained motivation and athletic performance (21, 22).

The ability to effectively control one's body in space was identified as a key skill in the sport analyzed. Most athletes reported a significant improvement in body awareness, an aspect that can translate into a substantial competitive advantage. As highlighted in previous studies, better body control is closely related to higher athletic performance (23, 24). This awareness, combined with increased maximum strength, represents a key element for athletic success in combat sports. Another relevant parameter that emerged from the present study was the increase in maximum strength perceived by the participants at the end of the 8 weeks. The perception of greater strength was confirmed by the test results, suggesting a close correlation between the improvement in physical abilities and the athletes' subjective perception (25–27). However, the study did not directly measure psychological motivation through self-reporting, nor did it monitor adherence to the training protocol through behavioral compliance measures. The absence of these data represents a limitation, as variations in motivation and compliance could significantly impact the observed results. These interpretations, however, should be viewed as indicative rather than conclusive due to the exploratory nature of the study.

### 4.1 Limitations

Despite its promising insights, this study has several limitations. The sample size was limited to 12 athletes, restricting the generalizability of the findings. In addition, only male athletes were included, which further limits the applicability of the results to the broader athletic population, particularly to female combat athletes. Most importantly, the absence of a control group prevents definitive conclusions about the causal effects of the training protocol. Although training compliance was observed, potential confounding factors such as nutrition, sleep quality, and additional unsupervised training sessions were not controlled, and these may have influenced the results. External factors, such as concurrent physical activities or psychological influences, could have contributed to the observed improvements. Future research should incorporate a control group and larger sample sizes to enhance the reliability and validity of conclusions. Moreover, the age range used introduces significant variability, as the physiological and psychological developmental differences between a 16-year-old athlete and a 27-year-old athlete can influence training adaptation, compliance levels, and reported perceptions (28). However, this variability did not compromise the study's objective, which was to examine the overall effectiveness of the protocol and its impact on the athletes' psychophysical aspects. Future studies could explore more homogeneous age groups to better isolate such differences. The statistical power of the analyses was deemed adequate based on the *a priori* power analysis, which indicated that a minimum of 10 participants was required. Nevertheless, given the relatively small sample size (*n* = 12), the study may still have been underpowered to detect smaller effects, which should be acknowledged as a limitation. Additionally, a more rigorous assessment of compliance with the training protocol is necessary, as the study did not track behavioral adherence or self-reported motivation levels. Without such data, it is challenging to determine the extent to which participants followed the prescribed regimen and whether their improvements can be directly attributed to the protocol itself. Another area for further investigation is the long-term sustainability of the observed strength gains. The study's duration was limited to 8 weeks, leaving open the question of whether continued training is necessary to maintain these improvements. Additionally, future studies should explore the impact of different training durations and intensities to identify optimal conditions for maximizing strength development. These findings align with previous research on training adaptation and sustainability in combat sports (29, 30). Further studies could explore how these elements interact with strength and body awareness to enhance performance in combat sports.

### 4.2 Future studies

The findings of this study suggest an interesting link between the strength training protocol and improvements in maximal strength and athletes' perceptions of body awareness and confidence. Additionally, the data supports the idea that integrating psychological factors into physical training programs could enhance athlete retention and motivation, particularly in younger athletes. For example, structured programs that improve perceived competence and self-efficacy may lead to greater enjoyment and persistence in sports participation (31, 32). These findings have practical applications for coaches aiming to balance physical conditioning with fostering positive self-perception among athletes. By emphasizing both physical and psychological benefits, the results provide a framework for designing comprehensive training programs that cater to the needs of combat athletes (33). However, these results should be considered preliminary due to the exploratory nature of the study and the absence of a control group. While the improvements observed are promising, further investigation is necessary to confirm the effectiveness of the protocol and to generalize the findings to a broader population.

## 5 Conclusion

This pilot study explored the effectiveness of a strength training protocol in a small cohort of Wushu Sanda athletes, highlighting improvements in maximal strength and body awareness. This protocol proved effective not only in increasing physical abilities but also in enhancing athletes' perception of controlling their bodies and the strength required to compete successfully. Future studies could build on the foundation laid by this research, expanding the sample of athletes involved and introducing a control group. It would also be useful to investigate the effects of protocols of different durations or intensities, to determine the optimal conditions to maximize benefits. Finally, further research could explore the interaction between strength, body awareness, and other psychological or technical factors that influence performance in combat sports. This study uniquely combines physical and psychophysical assessment, offering a more holistic view of athlete development. Future research with larger sample sizes, control groups, and experimental designs is necessary to validate these observations and establish causality. Moreover, incorporating psychological and motivational factors into strength training programs may play a critical role in optimizing training outcomes.

## Data Availability

The original contributions presented in the study are included in the article/Supplementary Material, further inquiries can be directed to the corresponding author.
